# Overexpression of c-Met increases the tumor invasion of human prostate LNCaP cancer cells *in vitro* and *in vivo*

**DOI:** 10.3892/ol.2014.2390

**Published:** 2014-07-28

**Authors:** YILI HAN, YONG LUO, JIAHUI ZHAO, MINGCHUAN LI, YONGGUANG JIANG

**Affiliations:** Department of Urology, Beijing Anzhen Hospital Affiliated to Capital Medical University, Beijing 100029, P.R. China

**Keywords:** prostate cancer, epithelial-mesenchymal transition, invasive potency, c-Met

## Abstract

c-Met is a transmembrane tyrosine kinase receptor that may be activated by hepatocyte growth factor, an inducer of epithelial-mesenchymal transition (EMT), to regulate the associated downstream gene expression. This process is critical to cell migration in normal and pathological conditions. In the present study, the function of c-Met in the process of EMT was investigated in prostate cancer. Initially, a c-Met stable expression cell line was constructed using EMT- and c-Met-negative LNCaP prostate cancer cells. Following the identification of c-Met in the transfected cells, the changes in EMT, phosphatidylinositol 3-kinase (PI3K) and extracellular signal-regulated kinase pathway biomarkers were determined by western blot analysis. MTT, soft agar and Transwell assays, and xenograft studies were used to investigate the effects of c-Met on the proliferation, migration and tumorigenicity of LNCaP cells. The results of the present study revealed downregulation of E-cadherin and upregulation of vimentin in LNCaP-Met cells. The results demonstrated that c-Met enhanced proliferation, migration and tumorigenicity capacity when compared with LNCaP and LNCaP-pcDNA3.1 cells. Furthermore, these EMT-like changes were mediated via the PI3K and mitogen-activated protein kinase signaling pathways. The present study clearly demonstrates a crucial function for c-Met in EMT development in prostate cancer. c-Met-targeted treatment may be an effective adjuvant therapy for improving survival rates in patients with prostate cancer.

## Introduction

c-Met is a transmembrane tyrosine kinase receptor that is phosphorylated and activated upon the binding of its ligand. The natural ligand for c-Met is hepatocyte growth factor (HGF)/scatter factor, which is produced by stromal and mesenchymal cells (1). The phosphorylation of c-Met activates downstream signaling pathways, which initiate biological effects in normal and pathological processes (2). Under physiological conditions, the HGF/c-Met axis is implicated in cell growth and differentiation, organ development and neovascularization, as well as tissue repair and regeneration (3). Accumulating evidence indicates that it is also important in cancer development (4,5).

A dysfunctional HGF/c-Met axis has been implicated in the development, invasion and angiogenesis of cancers (6), and an increasing number of studies have revealed the specific mechanism (7,8). Overexpression of c-Met occurs via paracrine and autocrine stimulation of HGF, which in turn stimulates cancer cell progression. The paracrine signaling pathway is activated by HGF secretion by stromal cells, whereas the autocrine signaling pathway is initiated by HGF that is generated from cancer cells. Generally, the paracrine signaling pathway of HGF is the major cause of c-Met expression, as not all tumor cells produce HGF (9). Previous studies have also shown that certain signaling pathways mediate increased cancer progression as result of the HGF/c-Met axis, typically the phosphoinositide 3-kinase (PI3K) and mitogen-activated protein kinase signaling pathways (10–13).

HGF induces the disassembly adhesion of epithelial cancer cells, thereby increasing motility and invasiveness, in a process termed epithelial-mesenchymal transition (EMT). EMT is important in cancer metastasis as it promotes the detachment of cancer cells from the primary tumor areas, leading to invasion of the vasculature and colonization of distant organs with secondary tumors (14).

Although EMT induced by HGF has been investigated in various types of cancer (15,16), the tumorigenic association between EMT and c-Met, particularly in prostate cancer, remains unclear. Previous studies have shown that a high level of c-Met expression is significantly implicated in prostate cancer aggressiveness and associated with a poor clinical outcome (17,18). Therefore, it is crucial that an in-depth understanding of the mechanism by which c-Met signaling regulates tumorigenic cell processes is gained in order to develop successful therapeutic strategies. In the present study, the EMT- and c-Met-negative LNCaP prostate cancer cells (19–22) were used to demonstrate that the overexpression of c-Met promotes the progression of prostate cancer via EMT.

## Materials and methods

### Cell culture

An EMT- and c-Met-negative human prostate cancer cell line, LNCaP, was cultured in Dulbecco’s modified Eagle’s medium (DMEM; Gibco-BRL, Carlsbad, CA, USA) supplemented with 10% fetal bovine serum (FBS; Gibco-BRL), 100 U/ml penicillin and 100 μg/ml streptomycin. Cells were cultured in a 5% CO_2_ humidified incubator at 37°C.

### Cell transfection

The full-length cDNA encoding human c-Met was amplified and the recombinant plasmid, pcDN- A3.1/c-Met was constructed (Invitrogen Life Technologies, Carlsbad, CA, USA). LNCaP cells at 75% confluency were transfected with pcDNA3.1/c-Met (LNCaP-Met cells) using Lipofectamine 2000 (Invitrogen Life Technologies) in a six-well plate for 48 h, and transfection with pcDNA3.1(−) alone was performed for the control group (LNCaP-pcDNA3.1 cells). Cells were then trypsinized and seeded onto a 10-cm dish. Stable LNCaP-Met and LNCaP-pcDNA3.1 cells were subjected to G418 selection (400 mg/ml), and after two weeks single-cell clones were selected and expanded. After G418 selection, five LNCaP-Met and three LNCaP-pcDNA3.1 cell clones were selected. Identification of c-Met was performed in all these cell clones and one clone in each group was selected to be used in the subsequent study.

### Immunofluorescence staining

To identify the c-Met expression, cells were fixed in 4% paraformaldehyde for 10 min and blocked with goat serum (Boshide Biotech Co. Ltd., Wuhan, China) for 30 min, then incubated at 37°C for 1 h with rabbit anti-c-Met primary antibody (1:150; Santa Cruz Biotechnology, Inc., Santa Cruz, CA, USA). Following three washes with phosphate-buffered saline, the cells were incubated with tetramethylrhodamine isothiocyanate-conjugated secondary antibody (1:100; Boshide Biotech Co. Ltd.) at 37°C for 1 h and stained with 4′,6-diamidino-2-phenylindole for five min. The fluorescence staining intensity and intercellular location were examined using a fluorescence-inverted microscope (Olympus BX51; Olympus Corporation, Tokyo, Japan).

### Western blot analysis

Total cellular proteins were extracted. A 30-μg protein extract was separated by 12% SDS-PAGE. The separated proteins were subsequently transferred to nitrocellulose membranes (Bio-Rad, Hercules, CA, USA), and the membrane was blocked with 5% non-fat milk in Tris-buffered saline for 1.5 h. The membranes were incubated for 1.5 h with the primary antibodies, polyclonal rabbit anti-human C-met, polyclonal rabbit anti-human p-C-met, polyclonal rabbit anti-human E-cadherin, polyclonal rabbit anti-human ERK and polyclonal rabbit anti-human AKT, and monoclonal mouse anti-human vimentin, monoclonal mouse anti-human p-ERK and monoclonal mouse anti-human p-AKT (Santa Cruz Biotechnology, Inc.) at various dilutions and hybridized for 1 h with secondary antibodies, horseradish peroxidase-conjugated goat anti-rabbit and goat anti-mouse IgG. (Boshide Biotech Co. Ltd.). Imaging was performed using the electrochemiluminescence detection system (Pierce Biotechnology, Inc., Rockford, IL, USA) and protein loading equivalence was assessed by the expression of glyceraldehyde 3-phosphate dehydrogenase.

### Cell proliferation assay

Cell growth was evaluated by MTT assay (Sigma-Aldrich, St. Louis, MO, USA). A total of 1×10^4^ cells/well were plated into 96-well tissue culture plates in DMEM containing 10% FBS to a final volume of 0.2 ml. Following incubation for 24, 48 and 72 h, cells were incubated with 20 μl MTT to a final concentration of 0.5 mg/ml at 37°C for 4 h. Next, the medium was removed and the precipitated formazan was dissolved by adding 200 μl of dimethyl sulfoxide (Sigma-Aldrich). Following agitation for 10 min, the samples were lysed and the absorbance was detected at a wavelength of 570 nm using a microplate reader (Model 450 Mioroplate Reader; Bio-Rad).

### In vitro transwell invasion assay

Polycarbonate filters (8 μm; Millipore, Billerica, MA, USA) were coated with 50 μg/cm^2^ of reconstituted Matrigel (Sigma-Aldrich). A total of 5×10^3^ cells in 300 μl of serum-free growth medium were then seeded into the upper chamber. Cells were incubated under normoxic conditions and allowed to migrate towards the complete growth medium for 24 or 48 h. Non-invading cells were removed mechanically using cotton swabs. The inserts were stained with crystal violet and the invasive cells on the lower surface were counted under a microscope (Olympus BX51; Olympus Corporation).

### Soft agar assay

Cells were resuspended in top agar medium (2 ml; DMEM containing 0.4% low-melting agarose and 10% FBS), and overlaid onto bottom agar medium (2 ml; DMEM containing 0.8% low melting agarose and 10% FBS) in six-well culture plates. After two-three weeks, colonies >0.1 mm in diameter were scored as positive. Colony formation efficiency was counted under a light microscope (Olympus BX51; Olympus Corporation).

### In vivo tumorigenicity assay

A total of 30 six-week-old male athymic nude mice, weighing 30 g (Shanghai Experimental Animal Center, Shanghai, China) were divided into three groups: LNCaP (control; n=10), LNCaP-pcDNA3.1 (control; n=10), and LNCaP-Met (test; n=10). The mice were maintained under specific pathogen-free conditions and provided with sterile food and water. Cells were harvested, washed and resuspended in serum-free DMEM at a concentration of 1×10^7^ cells/ml, and injected subcutaneously into the flank of each mouse. The tumor volume was measured weekly using the following formula: Tumor volume = (length × width^2^) × π/6. The mice were sacrificed after eight weeks. All experiments performed complied with the Guidelines of Animal Care of Capital Medical University (Beijing, China).

### Statistical analysis

All data are presented as the mean ± standard deviation. Student’s t-test was performed and P<0.05 was considered to indicate a statistically significant difference. All statistical tests were performed using SPSS version 11.0 software (SPSS, Inc., Chicago, IL, USA).

## Results

### Overexpression of c-Met in LNCaP-Met cells

**Figure 1:**
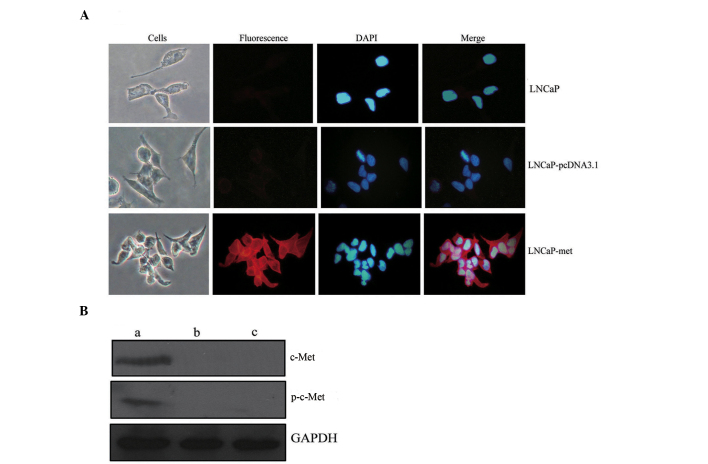
Overexpression of c-Met in LNCaP-Met cells. (A) Immunocytochemistry revealed marked red immunofluorescence staining in the membranes of LNCaP-Met cells when compared with LNCaP and LNCaP-pcDNA3.1 cells (tetramethylrhodamine staining, magnification, ×400). (B) Western blot analysis revealed the expression levels of c-Met and phospho-c-Met in (a) LNCaP-Met, (b) LNCaP and (c) LNCaP-pcDNA3.1 cells. DAPI, 4′,6-diamidino-2-phenylindole; GAPDH, glyceraldehyde 3-phosphate dehydrogenase.

To identify the increased expression of c-Met in the LNCaP-Met cell line, c-Met was examined using immunofluorescence staining and western blot analysis. Immunocytochemistry indicated significant immunofluorescence staining in the membrane of the LNCaP-Met cells, which, by contrast, was particularly weak in the membranes of the control LNCaP cells and the LNCaP-pcDNA3.1 cells (Fig. 1A). Increased expression of c-Met and phospho-c-Met was observed in the LNCaP-Met cells, however, no protein was detected in the control LNCaP or the LNCaP-pcDNA3.1 cells using western blot analysis (Fig. 1B). These results demonstrated that an LNCaP cell line with stable c-Met overexpression had been constructed and this was termed the LNCaP-Met cell line.

### Effect of c-Met expression on EMT-associated proteins

**Figure 2:**
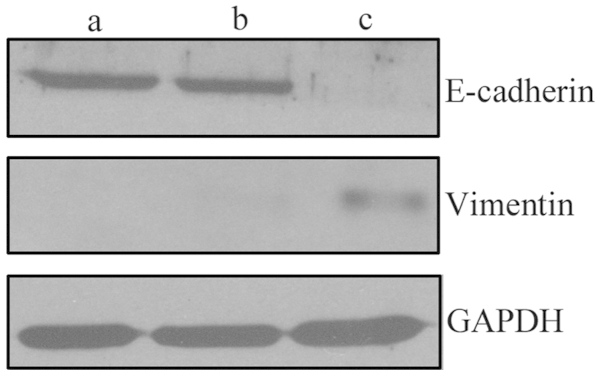
Changes in epithelial-mesenchymal transition-associated markers in three cell lines. The expression of E-cadherin and vimentin were determined by western blot analysis in (a) LNCaP-pcDNA3.1, (b) LNCaP and (c) LNCaP-Met cells. GAPDH, glyceraldehyde 3-phosphate dehydrogenase.

The development of EMT is characterized by the loss of epithelial markers, such as E-cadherin and the gain of mesenchymal markers, such as vimentin. To investigate changes in epithelial and mesenchymal markers, western blot analysis was used to determine the effect of c-Met on E-cadherin and vimentin in LNCaP-Met cells, compared with the control cells. In LNCaP-Met cells, the overexpression of c-Met downregulated E-cadherin, but upregulated vimentin (Fig. 2), which indicates that the overexpression of c-Met promotes an EMT phenotype in these cells.

### Effect of c-Met expression on the proliferation, migration and tumorigenicity of LNCaP-Met cells

**Figure 3:**
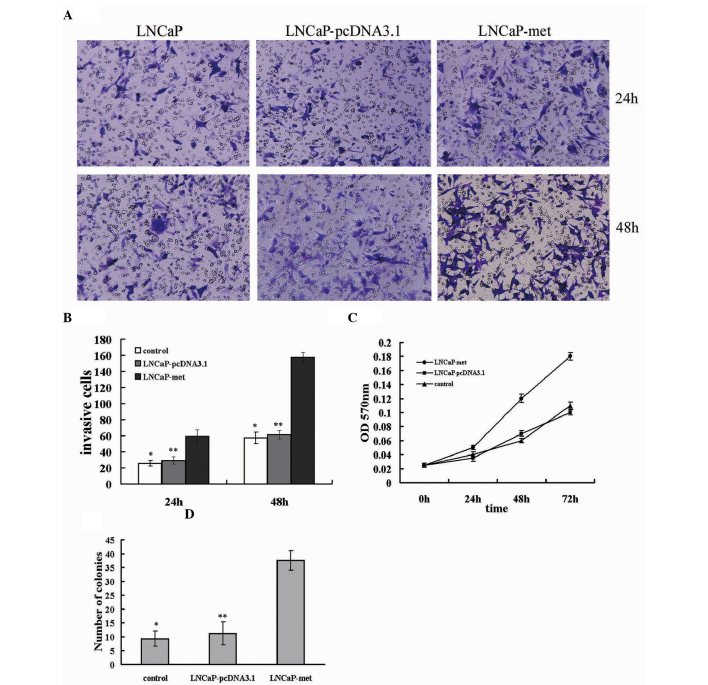
c-Met enhances the invasiveness of LNCaP-Met cells. (A and B) Transwell, (C) MTT and (D) soft agar assays indicated that c-Met increases the migration, proliferation and tumorigenicity of LNCaP-Met cells in contrast to the control LNCaP and LNCaP-pcDNA3.1 cells. ^*^P<0.05, LNCaP-Met vs. LNCaP cells; ^**^P<0.05, LNCaP-Met vs. LNCaP-pcDNA3.1 cells. OD, optical density. Crystal violet staining. Magnification, ×200.

c-Met promotes the migration of cancer cells, which is critical for metastasis. To determine the effect of increased c-Met expression on LNCaP-Met cell migration, cells migrating to the bottom of the insert were counted at 24 and 48 h. As shown in Fig. 3A and B, the migratory capacity was significantly increased, in a time-dependent manner, in LNCaP-Met cells when compared with LNCaP and LNCaP-pcDNA3.1 cells at 24 and 48 h (P<0.05).

To examine the effect of c-Met on LNCaP cell proliferation, the growth rate of the c-Met-transfected, control and parental LNCaP cells was determined. The LNCaP-Met cells exhibited a significantly higher growth rate than the LNCaP-pcDNA3.1 and LNCaP cells (P<0.05;Fig. 3C). However, no significant differences in cell growth rate were identified between the LNCaP-pcDNA3.1 and LNCaP cells (P>0.05; Fig. 3C).

The increased expression of c-Met resulted in a significant increase in the number of LNCaP-Met cell colonies formed when compared with the number of colonies formed in the parental LNCaP or LNCaP-pcDNA3.1 cells (P<0.05; Fig. 3D). By contrast, no significant differences were identified between the colony numbers of the LNCaP and LNCaP-pcDNA3.1 cells (P>0.05; Fig. 3D). Overall, these data indicate that EMT induced by c-Met overexpression stimulates LNCaP cell migration, proliferation and tumorigenicity.

### Effect of c-Met on tumorigenicity in vivo

**Figure 4:**
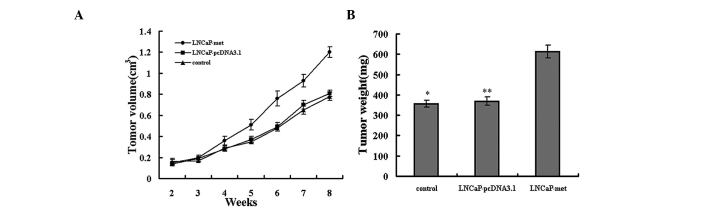
Xenograft studies in mice. (A) Tumors grew more rapidly in the LNCaP-Met cell group when compared with the other two groups. (B) The average tumor weight in the LNCaP-Met cell group was the largest after eight weeks, when compared with the other groups (P<0.05).^*^P<0.05, LNCaP-Met vs. LNCaP cells; ^**^P<0.05, LNCaP-Met vs. LNCaP-pcDNA3.1 cells.

To corroborate the observation that the upregulation of c-Met increased the invasive capacity of prostate cancer cells, the growth rates and metastatic behavior were analyzed *in vivo*. Tumor xenografts were established via subcutaneous injection into athymic nude mice. Subcutaneous tumors developed in five mice from the LNCaP-Met cells group, however, only grew in two mice from the LNCaP and LNCaP-pcDNA3.1 cell groups. Metastasis was not observed in any of the groups. The mean tumor volumes on days 14, 21, 28, 35, 42, 49 and 56 are shown in Fig. 4A. The volume of LNCaP and LNCaP-pcDNA3.1 cell tumors were significantly smaller than the LNCaP-Met cell tumors. On day 56 following implantation, the average weights of the LNCaP and LNCaP-pcDNA3.1 cell tumors were significantly lower than those of the LNCaP-Met cell tumors (Fig. 4B). These results indicate that EMT induction by overexpression of c-Met accelerates the growth of tumors *in vivo*.

### Effect of c-Met expression on extracellular signal-regulated kinase (ERK) and AKT signaling pathways

**Figure 5:**
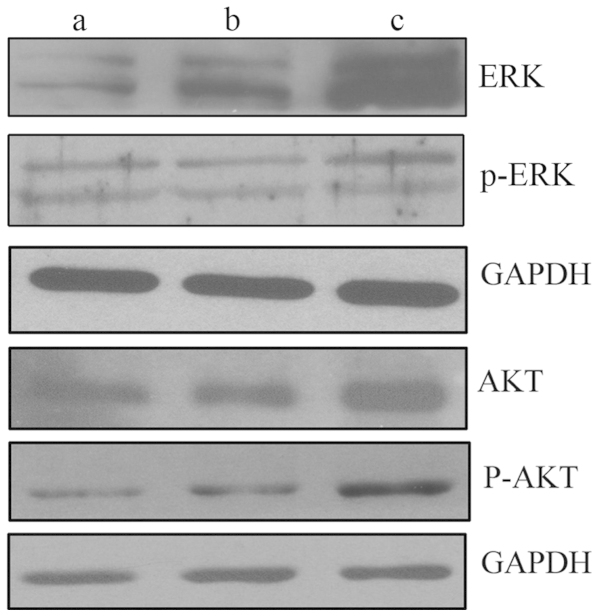
Western blot analysis of phosphoinositide 3-kinase and mitogen-activated protein kinase pathways in LNCaP-Met cells. Proteins were extracted from (a) LNCaP-pcDNA3.1, (b) LNCaP and (c) LNCaP-Met cells and the expression levels of ERK, p-ERK, AKT, p-AKT were determined. ERK, extracellular signal-regulated kinase; p, phosphorylated; GAPDH, glyceraldehyde 3-phosphate dehydrogenase.

To investigate the mechanisms involved in c-Met-induced EMT development, the levels of ERK and AKT signaling pathway components were determined by western blot analysis. ERK and AKT are two typical signaling pathways of c-Met. The results indicated that the increased expression of c-Met promotes EMT by upregulating the levels of ERK, phospho-ERK, AKT and phospho-AKT in LNCaP-Met cells (Fig. 5).

## Discussion

EMT is a process during which cancer cells lose their epithelial phenotype and acquire a mesenchymal phenotype, thus promoting the loosening of intercellular adhesions, detachment from the tumor mass and invasion of neighboring tissue, blood or lymph vessels, leading to the development of secondary tumors. Previous studies have indicated that EMT is implicated in cancer metastasis and invasion (23,25).

A number of factors, which induce EMT have been identified, including transforming growth factor-β, HGF, epidermal growth factor, fibroblast growth factor, platelet-derived growth factor, insulin-like growth factor, microRNA, hypoxia as well as transcription factors (25–27). Among these factors, EMT induction by the HGF/c-Met axis is essential in certain types of cancer (28–31), however, to the best of our knowledge, the role of c-Met in the progression of prostate cancer has not yet been reported.

In the present study, the effect of c-Met on the invasiveness of human prostate cancer was examined in LNCaP cells *in vitro* and *in vivo*. The results clearly demonstrated that by inducing EMT, c-Met overexpression enhances the invasion, migration and proliferation capability of LNCaP cells.

c-Met stable expression cell lines were constructed in c-Met- and HGF-negative LNCaP prostate cancer cells by cell transfection. Western blot analysis revealed that the expression of phospho-c-Met, as well as c-Met, was enhanced, which indicates that c-Met may be activated in an HGF-independent manner. A similar study demonstrated that c-Met may be activated via an HGF-independent signaling pathway, following transfection, in lung cancer (32). In addition, c-Met and HGF may be transactivated by the mutant of epidermal growth factor receptor (EGFR), also termed EGFRvIII (33). Furthermore, recent studies have demonstrated that c-Met activation occurs in the absence of ligand binding. Integrin activation, plexins, CD44, certain G protein-coupled receptors and other receptor tyrosine kinases have all been implicated in c-Met activation without a requirement for HGF binding (34).

Furthermore, activation of c-Met was found to activate the downstream signaling pathways PI3K and ERK, which resulted in the downregulation of E-cadherin and upregulation of vimentin. E-cadherin downregulation is regarded as a characteristic change of EMT (35). As intercellular adhesions are critical for the maintenance of the epithelial phenotype, the downregulation of E-cadherin (an essential component for adherent junctions) results in abnormal differentiation and the loss of cell polarity, which ultimately facilitates EMT (36). In addition, *in vitro* and *in viv*o studies demonstrated that the LNCaP-Met cell lines exhibited the greatest proliferative, migratory and tumorigenic potential, indicating a potential association between c-Met-mediated signaling pathways, EMT and prostate cancer aggressiveness. These results are consistent with a study showing that the acquisition of EMT characteristics is associated with the upregulation of c-Met mRNA and increased responsiveness to HGF in breast cancer (37).

To investigate the mechanism of EMT induction involving c-Met, the PI3K and ERK signaling pathways were examined. The two signaling pathways are considered to be typical c-Met-mediated signaling pathways (38) as well as EMT signaling pathways (39). Activation of the PI3K signaling pathway is associated with cell motility, whereas the ERK signaling pathway regulates cell proliferation and differentiation (40). The results of the present study revealed that AKT, ERK, phospho-AKT and phospho-ERK expression increased, indicating a molecular connection between EMT and c-Met. However, the mechanism at the transcriptional level requires further investigation.

Abnormal c-Met activation may occur in certain cancer types due to gene amplification, mutation or transactivation (41). However, c-Met overexpression as a result of upregulation at the transcriptional level is predominant in the majority of human malignancies (42). In the present study, it was found that the overexpression of c-Met signaling and the subsequent induction of EMT is potentially a common phenomenon in prostate cancer cells. Furthermore, EMT increased prostate cancer cell invasiveness *in vitro* and *in vivo*. All of these results demonstrated the potential association between c-Met, EMT and invasiveness in prostate cancer. Similarly, in other solid human tumors, various studies have shown that c-Met-mediated signaling activation drives EMT and cancer cell migration, invasion and metastasis (43–45).

In conclusion, the present study demonstrates that EMT induced by c-Met expression is involved in prostate cancer metastasis. Through the process of EMT, LNCaP prostate cancer cells acquire an increased proliferative, migrative and tumorigenic capability. Thus far, the role of c-Met in the progression and metastasis of numerous cancers has been described and a number of c-Met inhibitors have been investigated in clinical trials (46–48). Therefore, in addition to the transitional prognostic and predictive value, c-Met may present a promising therapeutic target in the fight against prostate cancer.

## References

[1] Jeffers M, Rong S, Vande Woude GF (1996). Enhanced tumorigenicity and invasion-metastasis by hepatocyte growth factor/scatter factor-met signaling in human cells concomitant with induction of the urokinase proteolysis network. Mol Cell Biol.

[2] Ponzetto C, Bardelli A, Zhen Z (1994). A multifunctional docking site mediates signaling and transformation by the hepatocyte growth factor/scatter factor receptor family. Cell.

[3] Birchmeier C, Gherardi E (1998). Developmental roles of HGF/SF and its receptor, the c-Met tyrosine kinase. Trends Cell Biol.

[4] Bhardwaj V, Cascone T, Cortez MA (2013). Modulation of c-Met signaling and cellular sensitivity to radiation: potential implications for therapy. Cancer.

[5] Gibney GT, Aziz SA, Camp RL (2013). c-Met is a prognostic marker and potential therapeutic target in clear cell renal cell carcinoma. Ann Oncol.

[6] Knudsen BS, Vande Woude G (2008). Showering c-Met dependent cancers with drugs. Curr Opin Genet Dev.

[7] De Bacco F, Luraghi P, Medico E (2011). Induction of MET by ionizing radiation and its role in radioresistance and invasive growth of cancer. J Natl Cancer Inst.

[8] Qian LW, Mizumoto K, Inadome N (2003). Radiation stimulates HGF receptor/c-Met expression that leads to amplifying cellular response to HGF stimulation via upregulated receptor tyrosine phosphorylation and MAP kinase activity in pancreatic cancer cells. Int J Cancer.

[9] Graveel CR, Tolbert D, Vande Woude GF (2013). MET: a critical player in tumorigenesis and therapeutic target. Cold Spring Harb Perspect Biol.

[10] Luraghi P, Schelter F, Krüger A, Boccacciol C (2012). The MET oncogene as a therapeutical target in cancer invasive growth. Front Pharmacol.

[11] Feng Y, Thiagarajan PS, Ma PC (2012). MET signaling: novel targeted inhibition and its clinical development in lung cancer. J Thorac Oncol.

[12] Li B, Torossian A, Sun Y, Du R, Dicker AP, Lu B (2012). Higher levels of c-Met expression and phosphorylation identify cell lines with increased sensitivity to AMG-458, a novel selective c-Met inhibitor with radiosensitizing effects. Int J Radiat Oncol Biol Phys.

[13] Seiden-Long I, Navab R, Shih W (2008). Gab1 but not Grb2 mediates tumor progression in Met overexpressing colorectal cancer cells. Carcinogenesis.

[14] Chung JY, Davis JA, Price BD (2011). Competitive enhancement of HGF-induced epithelial scattering by accessory growth factors. Exp Cell Res.

[15] Ogunwobi OO, Liu C (2011). Hepatocyte growth factor upregulation promotes carcinogenesis and epithelial-mesenchymal transition in hepatocellular carcinoma via Akt and COX-2 pathway. Clin Exp Metastasis.

[16] Previdi S, Maroni P, Matteucci E, Broggini M, Bendinelli P, Desiderio MA (2010). Interaction between human-breast cancer metastasis and bone microenvironment through activated hepatocyte growth factor/Met and β-catenin/Wnt pathways. Eur J Cancer.

[17] Pisters LL, Troncoso P, Zhau HE, Li W, von Eschenbach AC, Chung LW (1995). c-met proto-oncogene expression in benign and malignant human prostate tissues. J Urol.

[18] Cecchi F, Rabe DC, Bottaro DP (2010). Targeting the HGF/Met signaling pathway in cancer. Eur J Cancer.

[19] Luo Y, He DL, Ning L (2006). Expression of ‘epithelial-mesenchymal transition’ associated proteins in prostate cancer cell lines with different metastatic potentials and its significance. Zhonghua Nan Ke Xue.

[20] McKeithen D, Graham T, Chung LW, Odero-Marah V (2010). Snail transcription factor regulates neuroendocrine differentiation in LNCaP prostate cancer cells. Prostate.

[21] Wang Y, Yue D, Li K, Liu YL, Ren CS, Wang P (2010). The role of TRPC6 in HGF-induced cell proliferation of human prostate cancer DU145 and PC3 cells. Asian J Androl.

[22] Tate A, Isotani S, Bradley MJ, Sikes RA, Davis R, Chung LW, Edlund M (2006). Met-independent hepatocyte growth factor-mediated regulation of cell adhesion in human prostate cancer cells. BMC Cancer.

[23] Książkiewicz M, Markiewicz A, Zaczek AJ (2012). Epithelial-mesenchymal transition: a hallmark in metastasis formation linking circulating tumor cells and cancer stem cells. Pathobiology.

[24] Voutsadakis IA (2012). The ubiquitin-proteasome system and signal transduction pathways regulating Epithelial Mesenchymal transition of cancer. J Biomed Sci.

[25] Matsuoka J, Yashiro M, Doi Y (2013). Hypoxia stimulates the EMT of gastric cancer cells through autocrine TGFβ signaling. PLoS One.

[26] Luo Y, He DL, Ning L (2006). Over-expression of hypoxia-inducible factor-1α increases the invasive potency of LNCaP cells in vitro. BJU Int.

[27] Lamouille S, Subramanyam D, Blelloch R, Derynck R (2013). Regulation of epithelial-mesenchymal and mesenchymal-epithelial transitions by microRNAs. Curr Opin Cell Biol.

[28] Davidson B, Tropé B, Reich R (2012). Epithelial-mesenchymal transition in ovarian carcinoma. Front Oncol.

[29] Hamada S, Satoh K, Masamune A, Shimosegawa T (2012). Regulators of epithelial mesenchymal transition in pancreatic cancer. Front Physiol.

[30] Talbot LJ, Bhattacharya SD, Kuo PC (2012). Epithelial-mesenchymal transition, the tumor microenvironment, and metastatic behavior of epithelial malignancies. Int J Biochem Mol Biol.

[31] Leopold PL, Vincent J, Wang H (2012). A comparison of epithelial-to-mesenchymal transition and re-epithelialization. Semin Cancer Biol.

[32] Navab R, Liu J, Seiden-Long I (2009). Co-overexpression of Met and hepatocyte growth factor promotes systemic metastasis in NCI-H460 non-small cell lung carcinoma cells. Neoplasia.

[33] Garnett J, Chumbalkar V, Vaillant B (2013). Regulation of HGF expression by ΔEGFR-mediated c-Met activation in glioblastoma cells. Neoplasia.

[34] Varkaris A, Gaur S, Parikh NU (2013). Ligand-independent activation of MET through IGF-1/IGF-1R signaling. Int J Cancer.

[35] Nurwidya F, Takahashi F, Murakami A, Takahashi K (2012). Epithelial mesenchymal transition in drug resistance and metastasis of lung cancer. Cancer Res Treat.

[36] Shimada S, Mimata A, Sekine M (2012). Synergistic tumour suppressor activity of E-cadherin and p53 in a conditional mouse model for metastatic diffuse-type gastric cancer. Gut.

[37] Jahn SC, Law ME, Corsino PE (2012). An in vivo model of epithelial to mesenchymal transition reveals a mitogenic switch. Cancer Lett.

[38] Martin TA, Mason MD, Jiang WG (2011). Hepatocyte growth factor signaling in cancer metastasis. Curr Signal Transduct Ther.

[39] Thiery JP, Acloque H, Huang RY, Nieto A (2009). Epithelial-mesenchymal transitions in development and disease. Cell.

[40] Gentile A, Trusolino L, Comoglio PM (2008). The Met tyrosine kinase receptor in development and cancer. Cancer Metastasis Rev.

[41] Birchmeier C, Birchmeier W, Gherardi E, Vande Woude GF (2003). Met, metastasis, motility and more. Nat Rev Mol Cell Biol.

[42] Benvenuti S, Comoglio PM (2007). The MET receptor tyrosine kinase in invasion and metastasis. J Cell Physiol.

[43] Torres KE, Zhu QS, Bill K (2011). Activated MET is a molecular prognosticator and potential therapeutic target for malignant peripheral nerve sheath tumors. Clin Cancer Res.

[44] Lengyel E, Prechtel D, Resau JH (2005). C-Met overexpression in node-positive breast cancer identifies patients with poor clinical outcome independent of Her2/neu. Int J Cancer.

[45] Zhou AX, Toylu A, Nallapalli RK (2011). Filamin a mediates HGF/c-MET signaling in tumor cell migration. Int J Cancer.

[46] Inagaki Y, Qi F, Gao J (2011). Effect of c-Met inhibitor SU11274 on hepatocellular carcinoma cell growth. Biosci Trends.

[47] Falchook GS, Fu S, Amin HM (2011). Phase I dose-escalation study of the oral selective C-Met inhibitor EMD 1204831 in patients with advanced solid tumours. Eur J Cancer.

[48] Yap TA, Olmos D, Brunetto AT (2011). Phase I trial of a selective c-MET inhibitor ARQ 197 incorporating proof of mechanism pharmacodynamic studies. J Clin Oncol.

